# DMS triggers apoptosis associated with the inhibition of SPHK1/NF-κB activation and increase in intracellular Ca^2+^ concentration in human cancer cells

**DOI:** 10.3892/ijmm.2013.1541

**Published:** 2013-10-30

**Authors:** KAN CHEN, QIUWEI PAN, YING GAO, XINYAN YANG, SHIBING WANG, MAIKEL P. PEPPELENBOSCH, XIANGDONG KONG

**Affiliations:** 1 Bio-X Center, College of Life Sciences, Zhejiang Sci-Tech University, Hangzhou, Zhejiang 310018, P.R. China; 2 Department of Gastroenterology and Hepatology, Erasmus MC University Medical Center, Rotterdam, 3000 CA, The Netherlands; 3 Department of Radiotherapy Oncology, The First Affiliated Hospital of Medical College, Xi’an Jiaotong University, Xi’an, Shaanxi 710061, P.R. China

**Keywords:** N,N-dimethyl-D-erythro-sphingosine, nuclear factor-κB pathway, intracellular calcium concentration, sphingosine kinase 1

## Abstract

N,N-Dimethyl-D-erythro-sphingosine (DMS) is known to induce cell apoptosis by specifically inhibiting sphingosine kinase 1 (SPHK1) and modulating the activity of cellular ceramide levels. The present study investigated the effects and the mechanism(s) of action of DMS in human lung cancer cells. We found that DMS dose-dependently suppressed cell proliferation and induced cell apoptosis in the human lung cancer cell line, A549. Mechanistically, treatment with DMS suppressed the activation of SPHK1 and nuclear factor-κB (NF-κB) p65, but increased intracellular [Ca^2+^]i in A549 cells. This study demonstrates that DMS triggers the apoptosis of human lung cancer cells through the modulation of SPHK1, NF-κB and calcium signaling. These molecules may represent targets for anticancer drug design.

## Introduction

**Figure 1:**
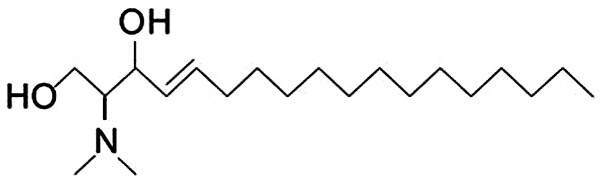
Chemical structure of N,N-dimethyl-D-erythro-sphingosine (DMS).

N,N-Dimethyl-D-erythro-sphingosine (DMS) (Fig. 1) is biologically derived from sphingosine and has been detected in several tissues (1). It has been reported that DMS can modulate phosphorylation events by inhibiting protein kinase (2) along with sphingosine kinase (3). DMS can inhibit the activity of sphingosine kinase 1 (SPHK1), resulting in an increase in ceramide levels and a decrease in sphingosine-1-phosphate (S1P) levels within the cell events involved in cell differentiation and apoptosis (4–6).

The nuclear factor-κB (NF-κB) family is involved in cellular responses to stimuli such as stress, cytokines, free radicals, ultraviolet irradiation, oxidized LDL and bacterial or viral antigens. Upon induction, it can transfer to the nucleus and stimulate the expression of various target genes, which play several crucial functions, including resistance to apoptosis and promotion of cell survival. Increased SPHK activity can alter the sphingolipid signal and NF-κB p65 expression and can eventually lead to the drug resistance of breast cancer cells (7). Inhibition of basal SPHK1 activity has been shown to induce apoptosis in A549 cells by interfering with constitutive NF-κB activity (8).

Calcium is an ubiquitous second messenger that controls a broad range of cellular functions. Previous studies have reported that DMS increases Ca^2+^ concentration within cells, including T lymphocytes, monocytes, astrocytes, neuronal cells (9–12) and HCT116 human colon cancer cells (13).

Lung cancer is a common aggressive malignancy worldwide with limited treatment options available. Experimental research has shown that SPHK1 inhibitor can significantly improve the curative effects of chemotherapy drugs on lung cancer cells, as well as other types of cancer cells (14–16). However, its mechanistic action has yet to be extensively investigated. In the present study, the effects of DMS on human lung cancer cells were examined. Further, the mechanistic link between DMS and SPHK1 expression, the NF-κB pathway and intracellular Ca^2+^ concentration in A549 cells were investigated.

## Materials and methods

### Reagents

DMS was purchased from Enzo Life Sciences (Enzo, New York, NY, USA) and was dissolved in dimethyl sulfoxide (DMSO). The final concentrations of DMSO were ≤0.1% in the drugs. Antibodies to SPHK1, NF-κB p65, poly-ADP-ribose polymerase (PARP) and GAPDH were purchased from Santa Cruz Biotechnology, Inc. (Santa Cruz, CA, USA). All other reagents were purchased from Sigma-Aldrich (St. Louis, MO, USA).

### Cell culture

The human lung cancer cell line, A549, was maintained in RPMI-1640 culture medium (Invitrogen, Carlsbad, CA, USA) supplemented with streptomycin (100 μg/ml) and penicillin (100 U/ml), glutamine (2 mM) and 10% (v/v) fetal bovine serum. The cells were grown at 37ºC in a humidified atmosphere containing 5% CO_2_.

### Morphological observations

Cells (5×10^5^/well) were plated in 6-well plates. After 12 h, the cells were exposed to serial dilutions of DMS (1, 2, 4 and 8 μmol/l) for the times indicated. The changes in cell morphology were observed under an inverted microscope at 48 h.

### Cell proliferation assay

Cells (6×10^3^/well) were transferred in 5 replicates to 96-well plates in 100 μl medium. All cells were incubated at 37ºC in 5% CO_2_ for 12 h to allow the cells to attach to the bottom of the wells. Serial dilutions of DMS (0.5, 1, 2, 4, 8 μmol/l) were added and the control group was supplemented with equal volumes of phosphate-buffered saline (PBS). At the culture times of 24, 48 and 72 h, the viability of the cells was analyzed after the addition of MTT and DMSO, respectively. Absorbance was determined using an enzyme mark instrument at a wavelength of 490 nm.

### Colony formation assay

The cells were washed, trypsinized and resuspended in culture medium, then counted and plated in 60-mm dishes (200 cells/dish) in triplicate and cultured in medium with serial dilutions of DMS, grown for 3 weeks, fixed with 10% methanol and stained with 2% crystal violet for 20 min. The dishes were washed and dried, and the colonies were counted to obtain a cloning efficiency for each DMS concentration.

### Analysis of cell apoptosis

The number of apoptotic cells was measured by staining the cell nuclei with Hoechst 33342 dye and the apoptotic cells were identified as those with condensed, fragmented nuclei. The cells were stained with 5 μl Hoechst 33342 (1 g/l) and incubated at 37ºC in the dark for 30 min following treatment with DMS at concentrations of 1 and 2 μmol/l for 24 and 48 h. Cell morphology was observed under a fluorescence microscope.

Flow cytometric analysis of apoptosis was performed by staining the cells with Annexin V-FITC and propidium iodide (PI). Cells (5×10^5^/well) were plated in 6-well plates and incubated overnight to attach to the bottom of the wells. Serial dilutions of DMS were then added and the control group was supplemented with equal volumes of PBS. After 48 h, the control or treated cells were resuspended in Annexin V-binding buffer, stained with fluorescein-conjugated Annexin V and PI (Annexin V-FITC kit; Becton Dickinson, Franklin Lakes, NJ, USA) and incubated at room temperature for 15 min. The cells stained only with Annexin V-FITC were used as the positive controls to set the apoptotic window, and the cells stained only with PI were used as the positive controls to set the necrotic window.

### DNA fragmentation analysis

Cells were plated in a 6-well plate and treated with serial dilutions of DMS. Twenty-four and 48 h later, the cells were collected by scraping and centrifuged at 600 × g for 10 min. The cells were then washed twice with PBS and DNA fragmentation was extracted using the Genome extraction kit (Generay Biotechnology, Shanghai, China) according to the manufacturer’s instructions. The DNA samples were subjected to electrophoresis on a 2% agarose gel and were then visualized under UV light after staining with ethidium bromide.

### Caspase-3 activity assay

Cells were plated in 6-well plates and treated with serial dilutions of DMS for 24 and 48 h. Subsequently, the cells were washed twice with cold PBS and lysed in lysis buffer (Beyotime Institute of Biotechnology, Shanghai, China) and placed on ice for 15 min. A sample of cytosolic protein was formed by centrifugation at 5,000 × g for 10 min and protein concentration was determined by the Bradford method. Cell extracts (30 μg protein) were incubated in reaction buffer containing Ac-DEVD-pNA (2 mM) at 37ºC for 2 h. Cleavage of the pNA fluorescence was detected using an enzyme mark instrument at an excitation wavelength of 405 nm. Caspase-3 activity was presented as units of fluorescence/(mg of protein × h).

### Quantitative RT-PCR (qRT-PCR)

Cells were washed with cold PBS and then harvested using TRIzol reagent (Invitrogen, Carlsbad, CA, USA); total RNA was extracted according to the manufacturer’s instructions. The extracted RNA was reverse transcribed into cDNA. The reverse transcription reaction system was 10 μl: 5X reaction buffer 2 μl, M-MuLV reverse transcriptase 0.5 μl, primer mix 0.5 μl, RNA 1 μg and DEPC added to a final volume of 10 μl. The samples were kept at 37ºC for 15 min and were then incubated at 98ºC for 5 min. The PCR components were set up as follows: 1 μl cDNA product, 12.5 μl master mix, 10 pmol/μl forward primer, 10 pmol/μl reverse primer and DEPC added to a final volume of 25 μl. PCR was performed with 30 cycles of denaturation: 5 min at 95ºC, 30 sec at 94ºC, 40 sec at 57ºC, and extension 30 sec at 72ºC. The primers were designed using primer 5 software. SPHK1 forward, 5′-gtt cca aga cac ctg cct cc-3′ and reverse, 5′-cac gca acc gct gac cat-3′; GAPDH forward, 5′-ggt gtg aac cat gag aag tat gac-3′ and reverse, 5′-tgg cag tga tgg cat gga ctg tg-3′. The amplified DNA fragment was separated on gel electrophoresis and analyzed by Applied Biosystems 7300 real-time PCR software.

### Western blot analysis

Cells were washed with cold PBS gently, supplemented with 100 μl/well cell lysis buffer (Beyotime Institute of Biotechnology) and placed on ice for 15 min. A sample of cytosolic protein was formed by centrifugation at 14,000 × g for 10 min and protein concentration were determined by the BCA method. The proteins (40 μg) were separated by 12% SDS-PAGE and then transferred onto PVDF membranes (Millipore, Bedford, MA, USA). The blots were blocked with 5% non-fat milk and then probed with primary antibodies (1:1,000 dilution) against the SPHK1 and GAPDH protein at 4ºC overnight. After washing, the membranes were incubated with secondary antibody (1:10,000 dilution) at room temperature. Antibodies were diluted in TBS containing 0.05% (v/v) Tween-20 and 5% BSA. Proteins were analyzed using the near infrared laser imaging system.

### Measurement of [Ca^2+^]i concentration

The intracellular [Ca^2+^]i concentration was measured using the fluorescent dye, Fluo-4/AM (Dojindo Laboratories, Kumamoto, Japan). The cells were treated with serial dilutions of DMS for 24 and 48 h, then resuspended in PBS containing 1% bovine serum and incubated for 30 min with 5 μM Fluo-4/AM in the dark. After washing with PBS, the Fluo-4/AM-labeled cells were observed under an inverted fluorescence microscope.

## Results

### Morphological alteration of A549 cells following treatment with DMS

**Figure 2:**
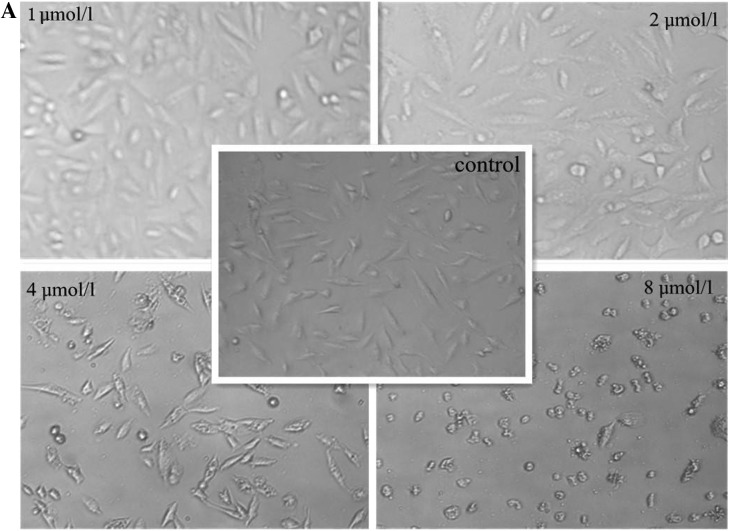

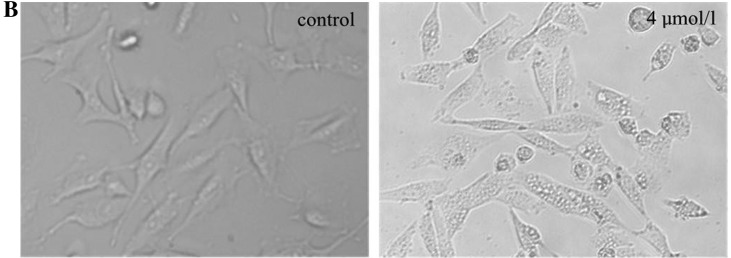
Changes in cell morphology were observed microscopically. (A) The number of viable cells of A549 markedly decreased after treatment with N,N-dimethyl-D-erythro-sphingosine (DMS) at the concentration of 4 μmol/l for 48 h. Original magnification, ×100. (B) Cell shrinkage and rounding was observed and vacuoles were also observed seen in the cytoplasm following treatment with DMS at concentrations of 0 and 4 μmol/l for 24 h. Original magnification, ×200.

**Figure 3:**
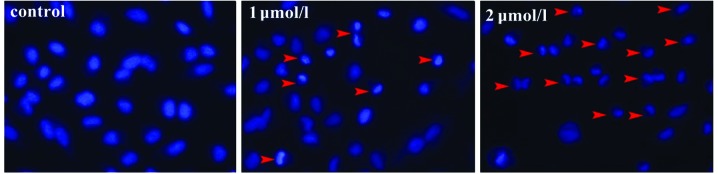
Staining with Hoechst 33342 after treatment with N,N-dimethyl-D-erythro-sphingosine (DMS) at concentrations of 0, 1 and 2 μmol/l for 24 h. Cells showed body shrinkage, nuclear fragmentation, nuclear dissolution and apoptotic bodies. Original magnification, ×320.

To explore the effects of DMS on the A549 cells, the cells were exposed to serial dilutions of DMS for 24 h. Cell morphology was observed under an inverted microscope. DMS dose-dependently altered the morphology of the A549 cells. At the concentration of 4 μmol/l, cell shrinkage and rounding was observed. Vacuoles were also observed in the cytoplasm (Fig. 2). The induction of apoptosis was further confirmed by Hoechst 33342 staining. Under a fluorescence microscope, with increasing concentrations of DMS, cell shrinkage, nuclear fragmentation, nuclear dissolution and apoptotic bodies were observed (Fig. 3).

### Cytotoxicity of DMS in A549 cells

**Figure 4:**
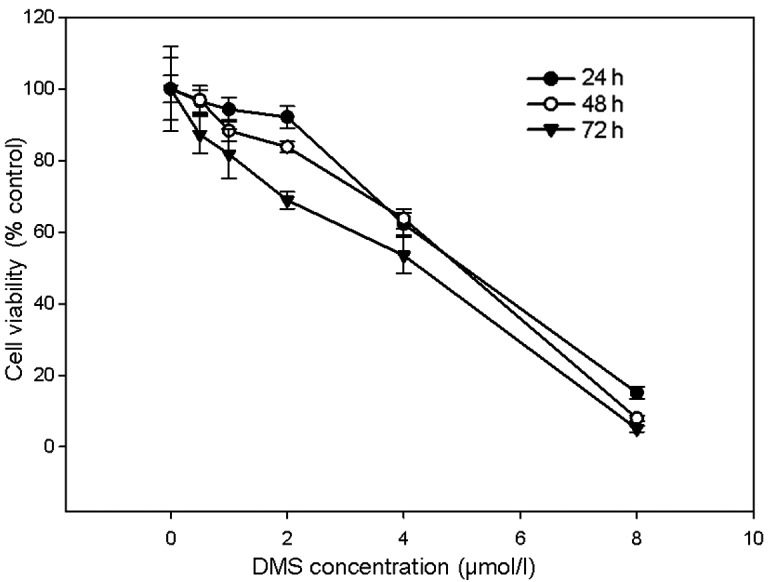
Cytotoxic activity of N,N-dimethyl-D-erythro-sphingosine (DMS) on A549 cells. Cells were treated with DMS at different concentrations for 24, 48 and 72 h. Cell viability was assayed by the MTT test. Shown is a representative result from at least 3 independent experiments.

The A549 cells were treated with serial dilutions of DMS for 24, 48 and 72 h. MTT assays were used to measure the cytotoxicity of DMS in the A549 cells. Treatment with DMS decreased the viability of the A549 cells in a dose- and time-dependent manner (Fig. 4). When DMS concentration reached 4 μmol/l, the cell survival rates were significantly decreased by 37.74±3.1, 36.25±2.82 and 46.5±5.11% (mean ± SD, n=6, P<0.01) at 24, 48 and 72 h, respectively (Fig. 4). The IC_50_ values for 24, 48 and 72 h were 4.864, 4.788 and 4.456 μmol/l respectively, calculated using SPSS 16.0 software.

### Inhibition of cell colony formation by DMS

**Figure 5:**
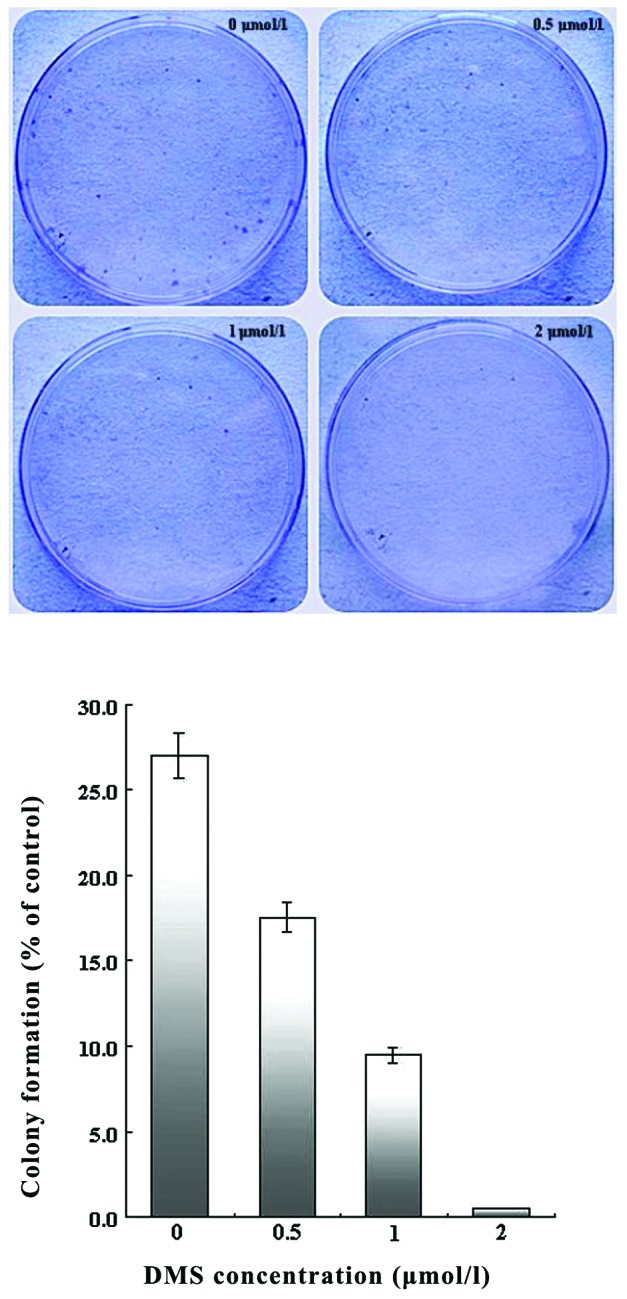
N,N-Dimethyl-D-erythro-sphingosine (DMS) inhibits A549 cell clonogenicity. Data are the means ± SD of 3 independent experiments.

To determine the effects of DMS on the ability of single cell proliferation, colony formation assay was performed. Colony formation efficiency was calculated with the number of visible colonies divided by the number of plated cells. Treatment with DMS suppressed colony formation in a dose-dependent manner. Once the concentration was >2 μmol/l, the growth of the A549 cells was almost completely inhibited, with a colony formation rate of <1% (Fig. 5).

### DMS induces cell apoptosis by activating the apoptotic signaling pathway

**Figure 6:**
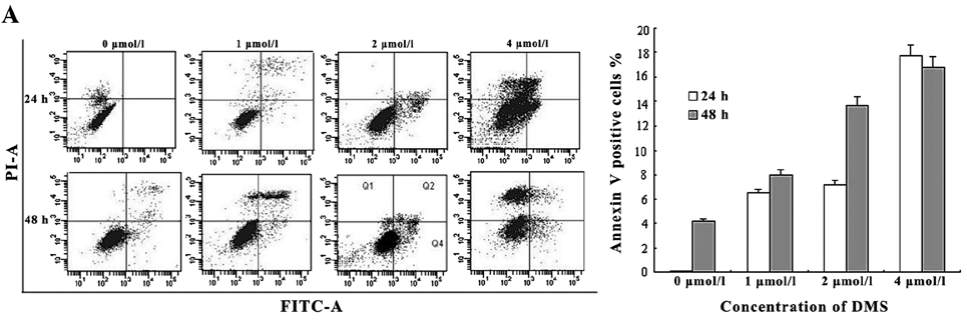

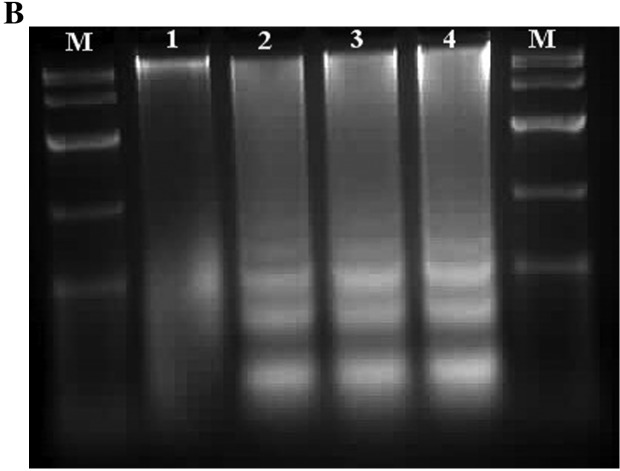
Apoptosis detection assay. (A) Cell apoptosis was observed by flow cytometry analysis after treatment with different concentrations of N,N-dimethyl-D-erythro-sphingosine (DMS) (0, 1, 2 and 4 μmol/l) for 24 and 48 h, respectively. (B) DNA fragmentation analysis after treatment with DMS for 24 and 48 h. M, DNA size markers; 1, untreated cells; 2, cells treated with 4 μmol/l DMS for 24 h; 3 and 4, cells treated with 2 and 4 μmol/l DMS for 48 h, respectively. Both panels are representative of 3 experiments.

The A549 cells were treated with various concentrations of DMS for 24 and 48 h, stained with Annexin V-FITC and PI, and analyzed by flow cytometry (Fig. 6A). FITC single-positive cells represent early apoptotic cells, FITC/PI double-positive cells represent apoptotic cells and PI single-positive cells represent dead cells. The percentages of apoptotic cells increased with the increasing DMS concentrations and with the prolonged exposure time. However, following treatment with 4 μmol/l DMS for 48 h, the percentage of dead cells reached 40.5% and the number of apoptotic cells in turn decreased. These results were further confirmed by DNA fragmentation assay. Treatment of the cells with DMS (4 μmol/l for 24 h, 2 and 4 μmol/l for 48 h) resulted in a classical laddering pattern, whereas no DNA laddering was observed in the controls (Fig. 6B).

**Figure 7:**
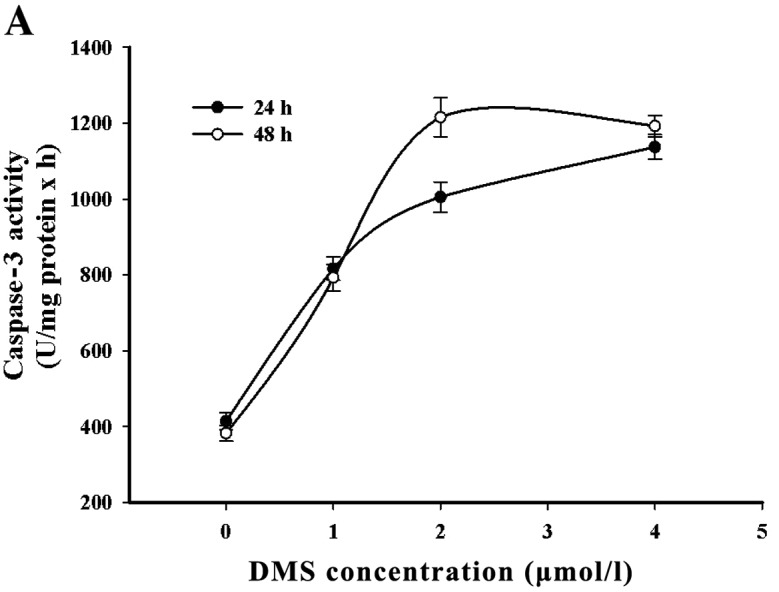

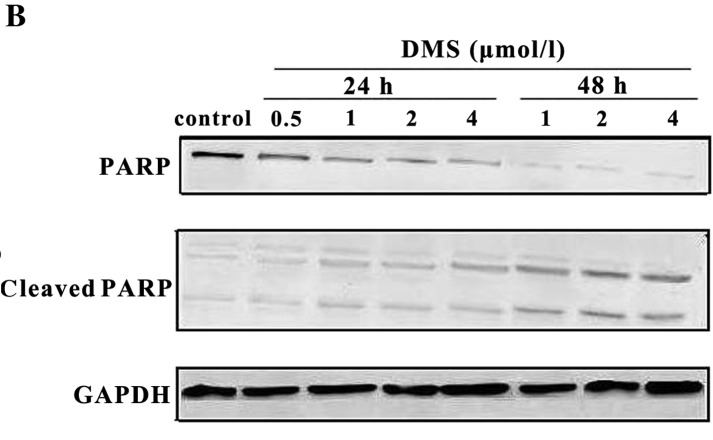
N,N-Dimethyl-D-erythro-sphingosine (DMS) activates the apoptosis signaling pathway. (A) Caspase-3 activity was measured in total lysates obtained from treated or untreated cells at 24 and 48 h after treatment. Data are means ± SD of three independent experiments. (B) A549 cells were treated with different concentrations of DMS for 24 and 48 h. Marked increase of PARP, apoptosis-related protein, was observed using western blot analysis. Data are from 3 independent experiments.

In order to investigate the signaling involved in DMS stimulation, caspase-3 activation in response to DMS was measured and the expression of PARP was analyzed by western blot analysis. The results revealed an increase in caspase-3 activity following treatment with DMS (1 μmol/l). It reached a plateau from 2 to 4 μmol/l. In addition, as a substrate of caspase-3, PARP was cleaved in a time- and concentration-dependent manner (Fig. 7).

### DMS suppresses gene expression of SPHK1

**Figure 8:**
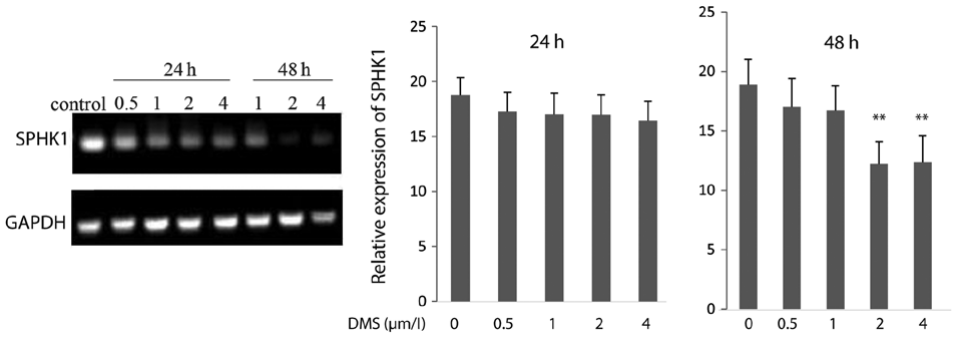
mRNA expression of specifically inhibiting sphingosine kinase 1 (SPHK1) in A549 cells. A549 cells were treated with various concentrations of N,N-dimethyl-D-erythro-sphingosine (DMS) for 24 and 48 h. SPHK1 mRNA expression was determined by RT-PCR and qRT-PCR. Data are the means ± SD of 3 independent experiments.

To investigate the effects of DMS on *SPHK1* gene expression, we analyzed the mRNA levels of *SPHK1* in A549 cells by RT-PCR. The results revealed that the mRNA levels of *SPHK1* were markedly downregulated following treatment with DMS at 2 and 4 μmol/l for 48 h. In addition, qRT-PCR revealed that the mRNA levels of *SPHK1* decreased by 35.28 and 34.64% when the cells were treated with 2 and 4 μmol/l DMS, respectively for 48 h (Fig. 8), indicating that DMS inhibits the expression of *SPHK1* at the transcriptional level.

### DMS inhibits SPHK1 and NF-κB activation

**Figure 9:**
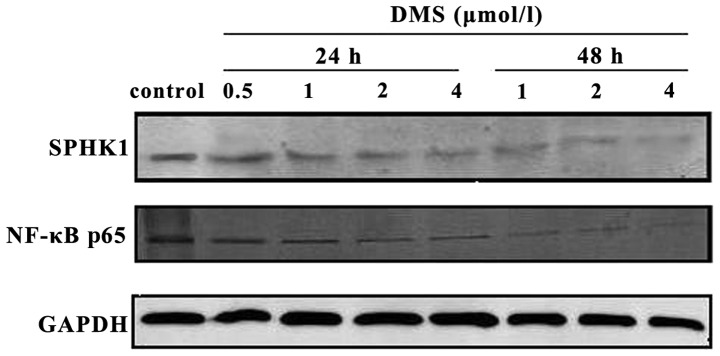
N,N-Dimethyl-D-erythro-sphingosine (DMS) specifically inhibits sphingosine kinase 1 (SPHK1) and the activation of the nuclear factor-κB (NF-κB) signaling pathway. Western blot assay was used to investigate the protein expressions of SPHK1 and NF-κB subunit p65 in A549 cells, treated with different concentrations of DMS for 24 and 48 h. Data are from 3 independent experiments.

Western blot analysis indicated that the expression of SPHK1 and the NF-κB p65 subunit decreased, with the increasing DMS concentrations and the prolonged treatment time. Therefore, the inhibition of NF-κB activity and SPHK1 expression may be responsible for the induction of cell apoptosis by DMS (Fig. 9).

### DMS increases intracellular Ca^2+^ concentration

**Figure 10:**
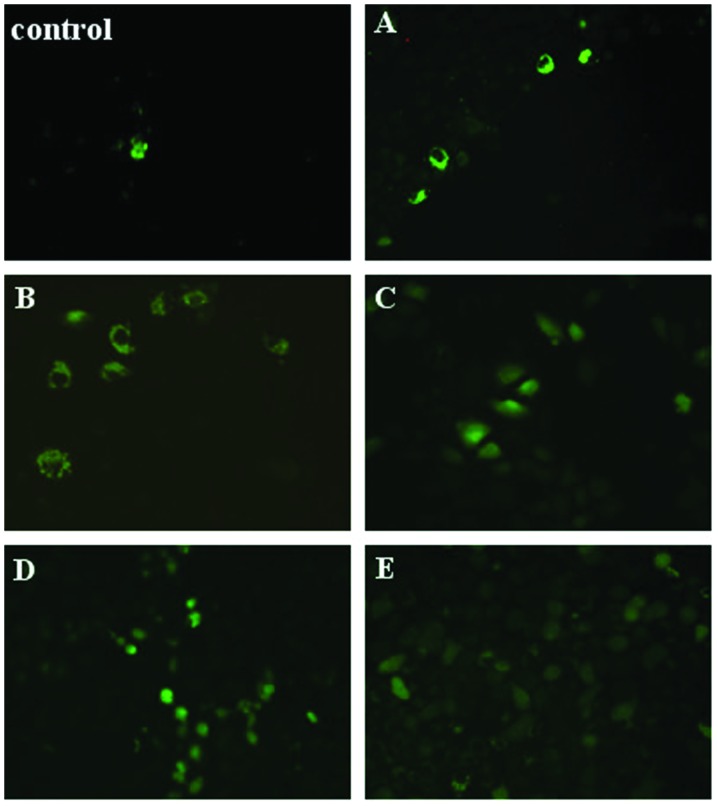
N,N-Dimethyl-D-erythro-sphingosine (DMS) increases intracellular Ca^2+^ concentration. (Control) Untreated cells; (A and B) cells treated with 2 and 4 μmol/l DMS for 24 h, respectively; (C–E) cells treated with 1, 2 and 4 μmol/l DMS for 48 h, respectively. Original magnification, ×200. Data are representative of 3 independent experiments.

The A549 cells were treated with various concentrations of DMS for 24 and 48 h and then incubated with Fluo-4-AM for 30 min. Fluo-4-AM can conjugate with [Ca^2+^]i and thus generate strong fluorescence in 405 nm after excitation light; therefore, intracellular [Ca^2+^]i levels can be indirectly visualized under an inverted fluorescence microscope. We observed that DMS increased intracellular [Ca^2+^]i concentrations in the A549 cells (Fig. 10).

## Discussion

Tumor progression depends mainly on the degree of cell proliferation and cell loss, and apoptosis is the main source of cell loss. SPHK1 is highly expressed in several types of tumor cells (approximately 2–3-fold higher) and its ability to prevent apoptosis has been extensively demonstrated (Table I) (17). There is evidence that the overexpression of SPHK1 contributes to cellular resistance to chemotherapy drugs (7). As an inhibitor of SPHK1, the anticancer properties of DMS have been widely investigated in preclinical models. The inhibition of tumor cell growth and migration by DMS has been reported (18–20) with a Ki value of 5 μmol/l (21,22). Moreover, the dose of DMS and tumor growth inhibition positively correlated in animal model of tumor-burdened nude mice.

The NF-κB signaling pathway in tumor biology has attracted considerable attention. It has been reported that cells expressing high levels of NF-κB are resistant to chemotherapy and radiotherapy (37). The inhibition of NF-κB activation sensitizes tumor cells to chemotherapy (38,39) and eventually lead to apoptosis. Consistently, in our study, we observed that triggering apoptosis in the A549 cells was associated with the inhibition of NF-κB activation. In fact, NF-κB is a calcium-dependent transcription factor (40). The disturbance of intracellular calcium triggers the elevation of reactive oxygen species in the mitochondria and leads to the translocation of NF-κB into the nucleus (41). A previous study reported that DMS increases the [Ca^2+^]i concentration in U937 and HCT116 cells (13). In the present study, we confirmed that DMS increased intracellular [Ca^2+^]i levels in A549 cells.

Billich *et al*(8) reported that the suppression of SPHK1 activation by DMS diminished NF-κB activity due to the reduced nuclear translocation of RelA (p65), resulting in spontaneous apoptosis in A549 cells. This is consistent with our experimental results. However, in our study, NF-κB activity was not increased, despite the increase in intracellular [Ca^2+^]i levels in A549 cells after treatment of DMS. These results suggest that other mechanisms may exist between the SPHK1 pathway and intracellular calcium signaling in terms of regulating NF-κB activity. When the SPHK1 pathway plays a major role, NF-κB activity may be diminished. By contrast, when intracellular calcium signaling plays a dominant role, NF-κB activity may be increased. However, the exact role of the SPHK1 pathway, calcium channels and the NF-κB signaling network in regulating the growth of cancer cells remains to be further elucidated.

## Figures and Tables

**Table I:** Summary of changes in SPHK1 expression in cancer tissues.

Tumor type	SPHK1 expression (Refs.)	Prognostic association (Refs.)	Associated with drug resistance (Refs.)
Breast	Increase (23,24)	Yes (23)	Yes (25)
Prostate	Increase (26)		Yes (27)
Ovary	Increase (28)		
Glioblastoma		Yes (29)	
Liver	Increase (30)		
Gastrointestinal	Increase (31)		
AML	Increase (32)		Yes (16)
Lung	Increase (33)		Yes (34)
Melanoma		Yes (35)	Yes (36)
